# AttCRISPR: a spacetime interpretable model for prediction of sgRNA on-target activity

**DOI:** 10.1186/s12859-021-04509-6

**Published:** 2021-12-13

**Authors:** Li-Ming Xiao, Yun-Qi Wan, Zhen-Ran Jiang

**Affiliations:** School of Computer Science and Technology, East China Normal University, Shanghai, 200062 China

**Keywords:** CRISPR, Attention mechanism, sgRNA design, Deep learning

## Abstract

**Background:**

More and more Cas9 variants with higher specificity are developed to avoid the off-target effect, which brings a significant volume of experimental data. Conventional machine learning performs poorly on these datasets, while the methods based on deep learning often lack interpretability, which makes researchers have to trade-off accuracy and interpretability. It is necessary to develop a method that can not only match deep learning-based methods in performance but also with good interpretability that can be comparable to conventional machine learning methods.

**Results:**

To overcome these problems, we propose an intrinsically interpretable method called AttCRISPR based on deep learning to predict the on-target activity. The advantage of AttCRISPR lies in using the ensemble learning strategy to stack available encoding-based methods and embedding-based methods with strong interpretability. Comparison with the state-of-the-art methods using WT-SpCas9, eSpCas9(1.1), SpCas9-HF1 datasets, AttCRISPR can achieve an average Spearman value of 0.872, 0.867, 0.867, respectively on several public datasets, which is superior to these methods. Furthermore, benefits from two attention modules—one spatial and one temporal, AttCRISPR has good interpretability. Through these modules, we can understand the decisions made by AttCRISPR at both global and local levels without other post hoc explanations techniques.

**Conclusion:**

With the trained models, we reveal the preference for each position-dependent nucleotide on the sgRNA (short guide RNA) sequence in each dataset at a global level. And at a local level, we prove that the interpretability of AttCRISPR can be used to guide the researchers to design sgRNA with higher activity.

**Supplementary Information:**

The online version contains supplementary material available at 10.1186/s12859-021-04509-6.

## Background

Clustered regularly interspaced short palindromic repeats (CRISPR)/CRISPR associated protein 9 (Cas9) systems are preferred over other biological research and human medicine technologies now, because of their efficiency, robustness, and programmability. Cas9 nucleases can be directed by sgRNA to introduce site-specific DNA double-stranded breaks in target, so to enable editing site-specific regions within the genome [1–3]. CRISPR/Cas9, to a large extent, have developed genetic therapies at the cellular level, albeit there are still severe medical disadvantages which have greatly hindered the further clinical application of the CRISPR/Cas9 systems. One of these disadvantages is due to unexpected insertion and deletion caused by the off-target effect [4–7]. To overcome this disadvantage, one solution is to engineer CRISPR/Cas9 with higher specificity. That's why more and higher specificity Cas9 variants, such as enhanced SpCas9 (eSpCas9(1.1)), Cas9-High Fidelity (SpCas9-HF1) [6, 8], hyper-accurate Cas9 (HypaCas9) [9], have been developed and bring a significant volume of experimental data, which means that researchers have to face the challenging of analyzing such huge and heterogeneous data. The activity of chosen sgRNA sequence determines the efficiency of genome editing, this fact indicates that it is meaningful to develop an efficient approach to predict sgRNA activity and even guide sgRNA design.

In practice, there have been several applications and toolkits applied in this task. In the earlier studies, the methods in silico are categorized into three types: (1) alignment-based, (2) hypothesis-driven, and (3) learning-based [10]. Recently, we noticed that the last type of method seems to be getting more attention because of huger and huger datasets [11]. The learning-based method is essentially a computational model built by machine learning algorithms, not only conventional machine learning but also deep learning. Some studies on HT_ABE and HT_CBE (two gene editing tools that grew out of CRISPR) have shown that deep learning-based models often outperformed conventional machine learning methods, when the number of sgRNA in the dataset reached a certain level [12–14]. Nevertheless, conventional machine learning algorithms, such as linear regression, logistic regression, and the decision tree, are often more interpretable due to the fewer parameters and clearer mathematical assumptions. In short, what was needed for the developers is to trade-off accuracy and interpretability. Some researchers consider the deep-learning models as a black box and believe they lack interpretability, motivated by the empirical assertion, they turn to build a model based on conventional machine learning to compete with state-of-the-art deep learning models [15]. On the other hand, input perturbation-based feature importance analysis becomes a preferred component to reveal the importance of features in deep learning models. Some use a sliding window of length 2 to extract dimeric as input and rank the position of dimeric by contribution to final output [16]. One regret is that their analysis cannot be on the independent nucleotide class exactly because of the processing of the input sgRNA sequence. Further, SHAP, one of the most prominent model explanation techniques, has been widely used to understand the decision made by the model. DeepHF, a deep learning-based model, uses Deep SHAP to reveal nucleotide contributions [17]. In our understanding, the method based on input perturbation often requires better generalization ability of the model (even for artificial ridiculous noise data).

In addition, the interpretability of existing models is all at the global level, and the result is a general pattern in the dataset that lacks analysis at the local level. They can explain which position has a great impact on the final decision of the model and which position-dependent nucleotide has a positive impact on the activity but not which structure causes the low activity of a certain nucleotide sequence and how to improve its activity with a few modifications. In light of the above, we believe it is critical to develop an effective model which can not only have good performance but also with good interpretability.

**Fig. 1:**
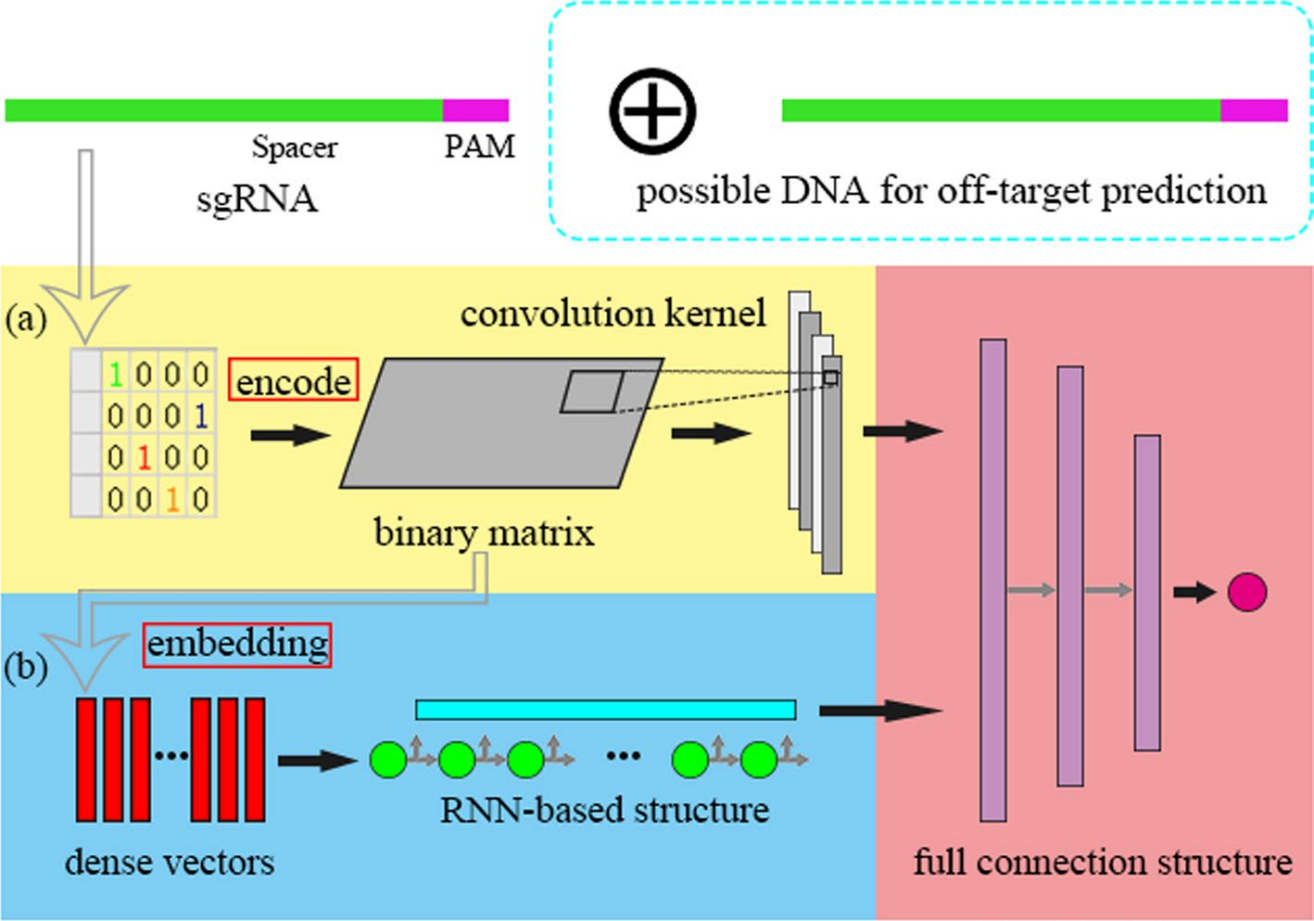
Two categories of deep learning models are used in sgRNA related tasks. **a** Model work in the spatial domain. In the spatial domain, the base sequence is encoded into a binary matrix (or a binary image). Since convolution has great advantages in extracting spatial features, CNN is an excellent tool in the spatial domain. **b** Model work in the temporal domain. In the temporal domain, the base sequence (represented by the binary matrix) is embedded into a sequence of high-dimensional vectors, in which the RNN performs better. In addition, we note that the last layers of these neural networks are usually full connection structures (not necessarily), which greatly increases the difficulty of understanding the decisions of these models

The deep neural network has shown its power in the study of CRISPR/Cas9 and its improved systems [11]. Most of the deep neural networks existing are the combination of recurrent neural network (RNN), convolutional neural network (CNN), fully connected neural network (FNN), and their variants. As shown in Fig. 1, we found that the deep learning models used in sgRNA on-target activity (even for off-target effect) prediction tasks in recent years can be divided into the following two categories according to the encoding approach of the sgRNA sequence (sgRNA-DNA sequence pair, for off-target effect prediction):

1. Methods in the spatial domain. Some previous studies have used the methods CNN-based to predict sgRNA on-target activity or off-target effect [10, 12, 18]. They process sgRNA base sequence inputs with the help of one-hot encoding idea. In other words, they regard it as two-dimensional image data, and use convolution layer to extract potential features in the spatial domain, it is worth noting that the bidirectional gated recurrent unit (BGRU, in short), an RNN variant, has been used after pooling layer of classic CNN network [19]. We explain that BGRU assists CNN to extract spatial features in one dimension, under this belief it belongs to this category.
2. Methods in the temporal domain. These methods are not used for an on-target activity or off-target effect prediction, until recently [16, 17, 20]. They consider the nucleotide (can also dimer or polymer) in the sgRNA sequence as a word, then a trainable matrix (could be either supervised or unsupervised) is used to project the word to the dense real-valued space. This technology is called embedding, which generates the base embedding. However, base embedding is not spatially interpretable (different from one-hot encode). Almost all of the methods in the temporal domain used in sgRNA on-target activity or off-target effect flatten the hidden state vector into a one-dimensional vector as the input of the fully connected layer. It is a pity that the temporal sequential dependency of the hidden state vector is rarely noticed.

Attention mechanism has demonstrated its power in NLP, Statistical Learning, Speech and Computer Vision. It makes the model tend to focus selectively on parts of the input, which helps perform the task effectively. Strictly speaking, we are not the first to bring attention mechanisms into this field. The most similar approach to ours is the work based on the transformer, a component based on the attention mechanism. They use it instead of RNN to improve the ability of temporal feature extraction, hence, enhance the performance of their model [16, 21]. In our work, the interpretability benefit from the attention mechanism is more focused. Our main contributions are as follows:

1. Present a novel deep-learning model, which can extract potential feature representation of sgRNA sequence in both spatial and temporal domain parallelly. Finally, the ensemble learning method is used to combine the two to achieve better performance than current state-of-the-art models.
2. Introduce the attention mechanism into our model. As a result, it does not need post hoc explanations techniques based on input perturbation to explain itself. It is intrinsically interpretable in both temporal and spatial domains. In the spatial domain, it's at the global level, while at the local level is in the temporal domain.
3. Through ablation analysis and testing a series of possible network structures, we find multiple components and strategies can improve the performance of AttCRISPR, which could outperform current state-of-the-art tools on the DeepHF dataset.

## Materials and methods

### Dataset

The dataset we used for training, validation and testing is the DeepHF dataset [17]. We extracted 55604, 58617, 56888 sgRNAs with activity (represented by insertion/deletion (indel)) for WT-SpCas9, eSpCas9(1.1) and SpCas9-HF1, respectively, from its source data, and use the same partition method to divide train set and test set.

### Sequence encoding and embedding

For encoding process, we use the complementary base to represent the original base in sgRNA. Further, we use one-hot encode strategy, that is to say, we encode each base in sgRNA into a four-dimensional vector (encode A,T,G,C into [1,0,0,0], [0,1,0,0], [0,0,1,0], [0,0,0,1], respectively), called one-hot vector. Then a sgRNA can be considered as a matrix $X_{oh} \in R^{l \times 4}$, named one-hot matrix (a little sparse, since a one-hot vector is zero in all but one dimension). We believe it is meaningful to regard *X*_*oh*_ as a binary image, therefore, it is used as an input of CNN, which performs well in the image field. Meanwhile, as mentioned above, the one-hot matrix is a little sparse.

To facilitate the training process, we can map each one-hot vector into a dense real-valued high-dimensional space, which is called embedding. In summary, at the matrix level, the formula is as follows:

1 $$X_{e} = X_{oh} E_{m}$$

where *X*_*e*_ named embedding matrix, $E_{m} \in R^{4 \times m}$ is a trainable transformational matrix, *m* refers to the dimension of embedding space. We believe it is also meaningful to regard nucleotides in the sgRNA sequence as words, and the sgRNA sequence itself as a sentence, guided by this belief *E*_*m*_ is the word embedding matrix and *X*_*e*_ is the sentence embedding in NLP. Therefore, *X*_*e*_ is used as an input of RNN (or its variant), which performs well in the NLP field.

As each element of *X*_*oh*_ is interpretable (representing whether there is a corresponding nucleotide type at the corresponding location), we call *X*_*oh*_ the spatial input, and the CNN that works on *X*_*oh*_ is the method in the spatial domain. On the other hand, different from *X*_*oh*_, *X*_*e*_ can only be explained in the first dimension (representing the embedding vector of corresponding nucleotide type), and embedding vector is difficult for humans to understand. That's why we call *X*_*e*_ the temporal input, and the RNN (or its variant) that works on *X*_*e*_ belongs to the method in the temporal domain.

### Neural network architecture

Based on the categorization above, we assume that the method in the spatial domain and the temporal domain are heterogeneous, which can satisfy the diversity premise of ensemble learning. Based on the assumption above and ensemble learning, we follow the stacking strategy to develop AttCRISPR which can extract potential feature representation of sgRNA sequence in both spatial and temporal domain parallelly. Further, we apply attention mechanisms in both spatial and temporal domains to enhance the interpretability of AttCRISPR.

### First-order preference and second-order preference

To introduce the neural network architecture of AttCRISPR, Let's define first-order preference and second-order preference for convenience. Taking a simple linear regression model as an example, for input $X \in R^{l}$, where *l* refers to the length of base sequences, predicted activity *y* is as follows:

2 $$y = AX$$

where $A \in R^{d}$. The total differential of *y* in Eq. (2) is as following:

3 $$dy = \sum\limits_{i}^{d} {A_{i} dX_{i} }$$

where *A*_*i*_ and *X*_*i*_ denotes the *i*-th dimension of the vector *X* and *A*, *A*_*i*_ indicates how dramatically the function changes as *X*_*i*_ changes in a neighborhood of *X*, in other words, the importance of *X*_*i*_. That's why we'll call *A* first-order preference in our paper. Specifically, we use a vector *A*_*i*_ to build the first-order original preference at position *i* within sgRNA sequence, and *X*_*i*_ is an embeddedness of the *i*-th feature, then *A* and *X* are two matrices. Further, the final result can be weighted by a trainable non-negative weight vector $W \in R^{l}$, as follow:

4 $$y = W \cdot AX^{T}$$

then we define $\tilde{A}$ as the first-order combine preference matrix (or just first-order preference), which means $\tilde{A}$ can be expressed linearly by *A* as follow:

5 $$\tilde{A} = BA$$

where the weight matrix $B \in R^{l \times l}$ is learned through attention mechanism, which we will call the second-order preference matrix in our paper as its calculation is based on first-order preference, it can explain how a particular pattern containing two nucleotides affects the base sequence. Then the predicted value can be expressed as:

6 $$y = W \cdot \tilde{A}X_{e}^{T}$$

### The method in spatial domain

**Fig. 2:**
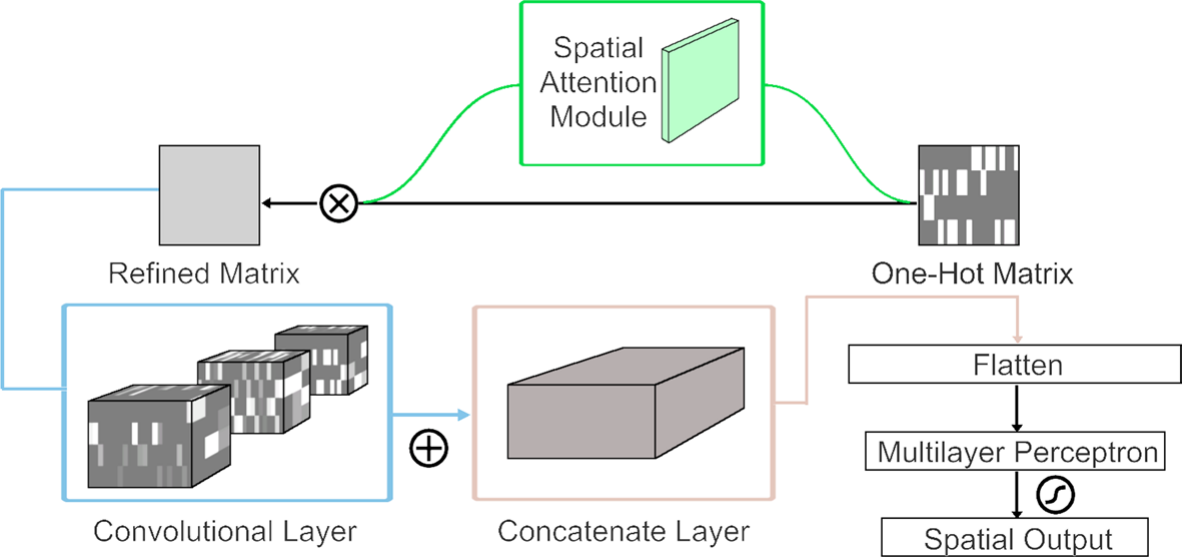
The architecture of spatial domain method in AttCRISPR. The input of the method is an encoded sgRNA sequence *X*_oh_, a 21 × 4 one-hot matrix. Then refine it through a spatial attention module, which could tell us the importance of a specific matrix element (or just say, pixel). A simple CNN followed is applied to extract potential feature representation of sgRNA sequence. In the last step, we flatten the output of the CNN into a one-dimensional vector and use a multilayer perceptron with a sigmoid activation function to achieve the spatial output *y*_*s*_

As demonstrated in Fig. 2, the method in the spatial domain relies on CNN. As previously mentioned, the sgRNA sequence has been encoded into a 21 × 4 one-hot matrix *X*_*oh*_, and we regard *X*_*oh*_ as a binary image. Then, convolution kernels with different sizes are used to extract potential spatial features just like other works have done in computer vision. According to the foregoing, the spatial attention module can be applied in our method [22], which has been used to improve the performance of CNN in vision tasks.

As shown in Additional file 1: Supplementary Figures Fig. S1, for a given one-hot matrix *X*_*oh*_, the spatial attention module generates a spatial attention matrix $A_{s} \in R^{l \times 4}$ with the same shape as *X*_*oh*_. Each element of *A*_*s*_ is constrained to a range of zero to one, implemented by a sigmoid function, which reflects the importance of the corresponding elements of *X*_*oh*_. The overall spatial attention process can be summarized as:

7 $$\left\{ \begin{gathered} X_{mc} = f^{3 \times 4} (X_{oh} ) \hfill \\ A_{s} = \sigma (f^{3 \times 2} ([AvgPool(X_{mc} );MaxPool(X_{mc} )])) \hfill \\ X_{rf} = A_{s} \otimes X_{oh} \hfill \\ \end{gathered} \right.$$

where $f^{p \times q}$ represents a convolution operation with the filter size of *p* × *q*, $p,q \in R^{{Z^{ + } }}$, *X*_*mc*_ is a multi-channel map generated by *X*_*oh*_, $\sigma ( \cdot )$ denotes the sigmoid function, $AvgPool( \cdot )$ denotes the average-pooling operation, $MaxPool( \cdot )$ denotes the max-pooling operation, ⊗ denotes element-wise multiplication. The spatial attention matrix *A*_*s*_ formally conforms to our proposed definition of first-order preference (each element of *X*_*oh*_ is multiplied by the corresponding element of *A*_*s*_), in other words, elements of *A*_*s*_ reveal how important the corresponding elements in *X*_*oh*_ are. We think it can reveal the preference of the scoring function at each position. For instance, following the encoding rules above, we train the spatial domain part of AttCRISPR with the WT-SpCas9 dataset. Then take the average of all spatial attention matrices, and the element in the first row and third column are closer to 1, which means when calculating the final score, G typically may have an important contribution at the first position within the sgRNA sequence. In fact, this corresponds to some early studies concerning the Human (hU6) promoter, which is believed to require G as the first nucleotide of its transcript [1–3].

### The method in the temporal domain

**Fig. 3:**
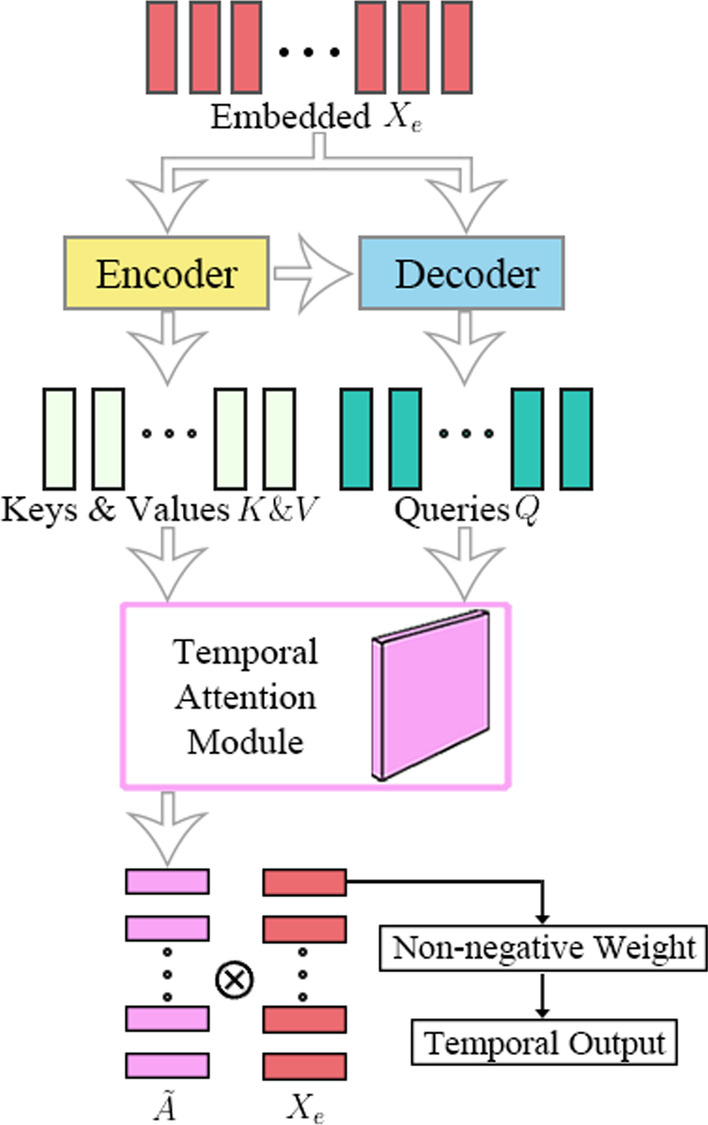
The architecture of temporal domain method in AttCRISPR. The input of the method is embedding the sgRNA sequence *X*_*e*_. Then Keys *K*, Values *V* and Queries *Q* are generated through a classic encoder-decoder structure which is needed by the temporal attention module. Next, the temporal attention module generates the first-order preference $\tilde{A}$. Each of the row vectors in matrix $\tilde{A}$ represents the base preference of sgRNA at the corresponding position, we use their dot product with the corresponding row vector in embedded *X*_*e*_ to build the score of the corresponding position. Hence, a full connection layer is used to weighted average them and achieve the temporal output *y*_*t*_

As shown in Fig. 3, the temporal domain part of AttCRISPR relies on the RNN (or its variant). As previously mentioned, we map each one-hot vector into a dense real-valued high-dimensional space following Eq. 1, which generates the embedded matrix *X*_*e*_. And we regard *X*_*e*_ as sequential data or temporal data. RNN (or its variant) has shown outstanding performance in the tasks with temporal data (for instance, NLP, sequential recommendation). That's why we prefer to use it to extract potential temporal features. To be precise, we prefer the architecture of encoder-decoder which has been proven to be effective in the Seq2Seq task. Two main differences we have to face are that sgRNA is not a natural language in the traditional sense, and we don't have to translate it to other sequences. To accommodate them, the embedded matrix *X*_*e*_ is used as input of both the encoder and decoder, and the output sequence of the decoder is used to build the first-order preference of sgRNA sequence $\tilde{A}$. As mentioned above, the predicted value *y* should satisfy Eq. 6.

On this basis, we apply the idea of attention mechanism which has been widely used in NLP tasks to AttCRISPR in the method of the temporal domain, and name it the temporal attention module. The temporal attention module satisfies the following equation

8 $$Attention(Q,K,V) = align(Q,K)V$$

where *Q*, *K*, *V* are queries, keys, and values matrix [21, 23].

As Additional file 1: Supplementary Figures Fig. S2 shows, in our attention module they are calculated by the following equation:

9 $$\left\{ \begin{gathered} K_{i} = Encoder(X_{ei} ,\theta_{E} ,K_{i - 1} ) \hfill \\ Q_{i} = Decoder(X_{ei} ,\theta_{D} ,Q_{i - 1} ) \hfill \\ V = K \hfill \\ \end{gathered} \right.$$

where vector *K*_*i*_, *Q*_*i*_ denotes the *i*-th row of the matrix *K* and *Q* accordingly, *Encoder*(·) and *Decoder*(·) are independent GRU units, $\theta_{E}$ and $\theta_{D}$ denote all the related parameters of GRU networks accordingly. In the actual implementation, we apply the bidirectional GRU networks for better performance, and for the sake of conciseness, we show a conventional GRU network here. The function *align*(·)is as follows:

10 $$B = align(Q,K)$$

11 $$B_{i} = softmax(Q_{i} K^{T} ) \otimes G_{i}$$

12 $$G_{ij} = \left\{ {\begin{array}{*{20}l} {\exp \left( {\frac{{(i - j)^{2} }}{ - 2\sigma }} \right),} \hfill & {\left| {i - j} \right| \le \sigma } \hfill \\ {0,} \hfill & {\left| {i - j} \right| > \sigma } \hfill \\ \end{array} } \right.$$

where matrix $B \in R^{l \times l}$ is the second-order preference we need, and vector *B*_*i*_ denotes the *i*-th row of the matrix *B*. $G \in R^{l \times l}$ is the damping matrix base on the Gaussian function. Since a simple belief that the closer the base is to the *i*-th position, the more influence it has on the *i*-th position, we use the damping matrix *G* to constrain the network learning. $\sigma$ represents a threshold of length, any base over this length from the position *i* is not considered to be affected. Further, if we think of the values matrix as a vector form of the first-order preference *A* in Eq. 5, we can reach the following equation:

13 $$\tilde{A} = BV$$

according to the above mentioned, the values matrix *V* comes from the hidden states of a bidirectional GRU network, which is usually hard to understand. While *B* is the second-order preference matrix obtained by the attention mechanism. We believe that the *j*-th dimension of *B*_*i*_, denoted as *B*_*ij*_, can reveal the effect of the base at position *j* on position *i* in the biological sense.

### Ensemble model following stacking strategy

Some indirect sgRNA features, which can't be obtained directly by deep learning, including position accessibilities of secondary structure, stem-loop of secondary structure, melting temperature, and GC content are strongly associated with sgRNA activity. It's worth noting that the hand-crafted biological features are not standardized in the work of others[17, 24]. Since the wide range of data distribution, we standardize it based on Z-Score.

Then we use a simple fully connected network to extract the indirect features, and call the output of the fully connected network *y*_*bio*_. As mentioned above, we assume that the method in the spatial domain and in the temporal domain can satisfy the diversity premise of ensemble learning. That's why we follow the stacking strategy, to integrate the methods in the spatial domain and the temporal domain. Specifically, the *y*_*bio*_, the spatial output *y*_*s*_ and the temporal output *y*_*t*_ we got earlier are concatenated, and then weighted averaging is performed through a full connection layer as follow:

14 $$y = W[y_{bio} ;y_{s} ;y_{t} ]$$

where *y* is the final prediction value of AttCRISPR, *W* is the weight learned by the full connection network. In the actual implementation, we freeze the network in the spatial domain and temporal domain firstly, in order to make our network focused on learning the weight *W*. Then the parameters of the entire network are adjusted in the fine tuning of AttCRISPR.

## Experiment design

Two different experiments are carried out in our work, which follow the same strategy as DeepHF. To be more specific, each set is shuffled and divided into three parts, 76.5%, 15%, and 8.5% of the relevant data were used as the training, test, and validation set respectively in a single experiment. The experiment is repeated ten times with the results recorded and averaged finally.

The first one is designed for the ablation analysis of AttCRISPR. We compare the performance of end2end methods (without any hand-crafted biological features) in both spatial and temporal domains. Furthermore, we test the ensemble method based on the same strategy to prove that the ensemble method in both spatial and temporal domains can significantly improve the performance.

The second experiment is designed to compare the performance of AttCRISPR with other current prediction methods. In order to make the comparison apples to apples, we reduce the dimensionality of the same hand-crafted biological features as DeepHF's, which has been shown to enhance the predictability of a deep-learning model greatly, with a multilayer perceptron. Then follow Eq. (14) to achieve the final prediction value. AttCRISPR (with the hand-crafted biological features) performs better on all three datasets than DeepHF.

Our baselines have comprehensive coverage of the methods tested in these datasets. In Table 1, we annotate some properties of these baselines (is/isn't neural models, is/isn't end2end models). All of the experiments were carried out in Python 3.6 using Keras 2.2.4 and one GeForce RTX 2080Ti Super was used for training and testing if needed.

**Table 1:** The main ideas of ANMDA and 6 published methods

Method	Neural	End2end	Description
CNN*	Yes	Yes	Naive CNN
RNN*	Yes	Yes	Bidirectional long short-term memory neural network
XGBoost*	No	Yes	Extreme Gradient Boosting regression tree
MLP*	Yes	Yes	Multilayer perceptron
DeepHF*	Yes	No	Bidirectional long short-term memory neural network (with hand-crafted biological features)
CRISPRpred#	No	No	A conventional machine learning pipeline
SpAC	Yes	Yes	Spatial AttCRISPR
TAC	Yes	Yes	Temporal AttCRISPR
EnAC	Yes	Yes	Ensemble AttCRISPR (without hand-crafted biological features)
StAC	Yes	No	Standard AttCRISPR

The method with superscript of * and # have been reported respectively [15, 17]. Especially, CRISPRpred takes another set of hand-crafted sequence-based features to improve performance

We design experiments to address the following questions:

1. In the absence of hand-crafted biological features, whether the stacking of methods in the spatial domain and temporal domain can get better performance than using these methods alone?
2. How does AttCRISPR perform compared to current state-of-the-art methods, covering both conventional machine learning and deep-learning models?
3. How can researchers understand the decisions made by AttCRISPR locally and globally, based on attention mechanisms?

## Model building and stacking

In Table 2, we list the performance of methods in the spatial or temporal domain and the stacking of methods. Temporal AttCRISPR, TAC for short, achieved Spearman correlation coefficients of 0.857, 0.844, 0.851 respectively in the above three datasets. Spatial AttCRISPR, SpAC for short, corresponds to 0.862, 0.854, 0.857. In the absence of hand-crafted biological features. Ensemble AttCRISPR achieves the best performance of our knowledge, corresponding to 0.868, 0.859, 0.862.

**Table 2:** Performance comparisons for different methods in the absence of hand-crafted biological features (take Spearman rank correlation coefficient and mean squared error as evaluation index)

Method	WT-SpCas9		eSpCas9(1.1)		SpCas9-HF1	
	Spearman	MSE (× 10^–3^)	Spearman	MSE (× 10^–3^)	Spearman	MSE (× 10^–3^)
XGBoost*	0.845	11.7	0.831	11.5	0.818	13.5
MLP*	0.842	11.7	0.846	10.5	0.844	11.2
CNN*	0.846	11.3	0.831	11.3	0.834	12.0
RNN*	0.856	10.4	0.849	10.2	0.851	10.6
TAC	0.857	10.3	0.844	10.5	0.851	10.7
SpAC	0.862	10.1	0.854	9.93	0.857	10.2
EnAC	**0.868**	**9.51**	**0.859**	**9.64**	**0.862**	**9.81**
CRISPRpred#	0.838	–	0.830	–	0.821	–

In addition, in Table 2, the performance of other methods without using hand-crafted biological features, are also recorded. Regardless of the method we developed, RNN, which can be categorized as the method in the temporal domain, is the most predictive with Spearman correlation coefficients of 0.856, 0.849, 0.851 [17]. It's obvious that the ensemble AttCRISPR is better at prediction (Additional file 1: Supplementary Figures Fig. S3(a–c)). Furthermore, the prediction ability of models could be boosted by the addition of other hand-crafted biological features, which can't be obtained directly by sequence information.

A further experiment is designed to compare the performance of standard AttCRISPR (hand-crafted biological features are used to improve the performance of ensemble AttCRISPR) and DeepHF, which is a current state-of-the-art method.

## Performance comparison

To validate the conclusion that integrating with hand-crafted biological features can improve the predictive performance of methods, we follow Eq. (14) to modify the ensemble method and design the control experiment using the same strategy. What's more, we compare the standard AttCRISPR and DeepHF (Table 3).

**Table 3:** Performance comparisons for the methods before and after integrating with hand-crafted biological features (take Spearman correlation coefficient as evaluation index)

Method	WT-SpCas9		eSpCas9(1.1)		SpCas9-HF1	
	Mean	SD (× 10^–3^)	Mean	SD (× 10^–3^)	Mean	SD (× 10^–3^)
RNN*	0.856	3.33	0.849	5.00	0.851	4.11
EnAC	0.868	2.66	0.859	4.66	0.862	3.19
DeepHF*	0.867	**2.37**	0.862	4.24	0.860	3.21
StAC	**0.872**	2.55	**0.867**	**3.71**	**0.867**	**2.65**

The method with superscript of * and # have been reported respectively [15, 17]. In the tables, we use the results reported in the relevant papers as the performance of the method directly

As shown in Table 3, in the absence of hand-crafted biological features, AttCRISPR has significant advantages over DeepHF in predictability. Further, integration with the hand-crafted biological features can also improve the performance of AttCRISPR, and achieve Spearman correlation coefficients of 0.872, 0.867 and 0.867 for WT-SpCas9, eSpCas9(1.1) and SpCas9-HF1, respectively. Meanwhile, DeepHF achieves 0.867, 0.862 and 0.860, respectively. After integrating with biological features, the performance gap between AttCRISPR and DeepHF is shortened, while AttCRISPR still has better performance (Additional file 1: Supplementary Figures Fig. S4). In addition, we also compare the standard deviation of data obtained in ten tests, which are also shown in Table 3. It reveals that AttCRISPR is more stable than DeepHF.

## Interpretability of the AttCRISPR

In the following sections, we will analyze the insight into the activity of sgRNA brought through the attention mechanism at both global and local levels to validate the attention module in the AttCRISPR can help us to understand the decisions.

### Global interpretability

**Fig. 4:**
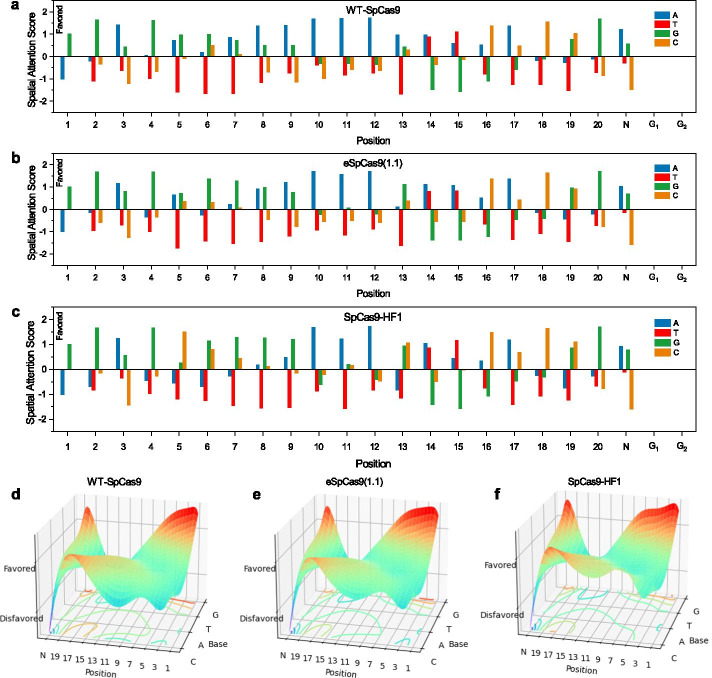
Preference for each position-dependent nucleotide on the sgRNA sequence. **a**–**c** Bars show the score of preference after standardization, and the higher the number, the more positive it is for the activity of sgRNA. The numbers below indicated the position of the nucleotides on-target DNA. **d**–**f** Preference surfaces for each position-dependent nucleotide fitted with Bézier surfaces. Each position-dependent nucleotide is a control point. The coordinates of the control points on the vertical axis represent the degree of preference, and the higher the position-dependent nucleotide corresponding to the control point is, the more positive it is for the activity of sgRNA. The contour plots at the bottom show the area of position-dependent nucleotides which have different contributions to the activity of sgRNA.

At the global level, an important question we expect AttCRISPR to answer is which nucleotide it prefers at each position on the sequence. In fact, this question has already been answered in detail with the DeepSHAP method [17]. While our method is not based on the post hoc explanations techniques and input perturbations, the only work we need to do is to get the first-order preference *A* generated by the attention module. Specifically, we use the first-order preference *A*_*s*_ generated by the spatial attention module instead of $\tilde{A}$ generated by the temporal attention module. The latter is in a higher dimensional dense space which makes it difficult to understand. In practice, we input every sgRNA into the spatial AttCRISPR, to obtain the *A*_*s*_ from the spatial attention module and take its mean value. Then we rescale it through Z-score to obtain a standardization value and the final result is shown in Fig. 4 and Additional file 2: Data S3.

As shown in Fig. 4, we captured the preference for each position-dependent nucleotide on the sgRNA sequence. The result revealed that A and G typically have a positive contribution to the activity of sgRNA, while T typically has a negative contribution. This agrees with the previous conclusion that when Cas9 is binding sgRNA, it prefers the one containing purines to pyrimidines [25]. In addition, global interpretability also pointed out that distinct from other nucleotides, G is strongly favored at position 20. This is consistent with the conclusions of several other reports [26, 27].

Furthermore, the preference of the nucleotide at the same position doesn't change dramatically with the Cas9 nucleases, while we still notice that compared with the other two datasets, C makes a more positive contribution to the activity of sgRNA with the SpCas9-HF1 especially in the position 5, which is evident in Fig. 4 (d-f).

The above discussion shows that, in the task of sgRNA activity prediction, the attention mechanism can help us understand the decision made by AttCRISPR and reveal insight into the activity of sgRNA.

### Local interpretability

At the local level, we analyze a case (consisting of three sgRNAs as  Additional file 1: Supplementary Tables Tab. S1 show), then we expect AttCRISPR to answer two important questions based on the local interpretability. First, how can we optimize a sgRNA to have more on-target activity? Second, what are the reasons for the low activity of the sgRNA?

**Fig. 5:**
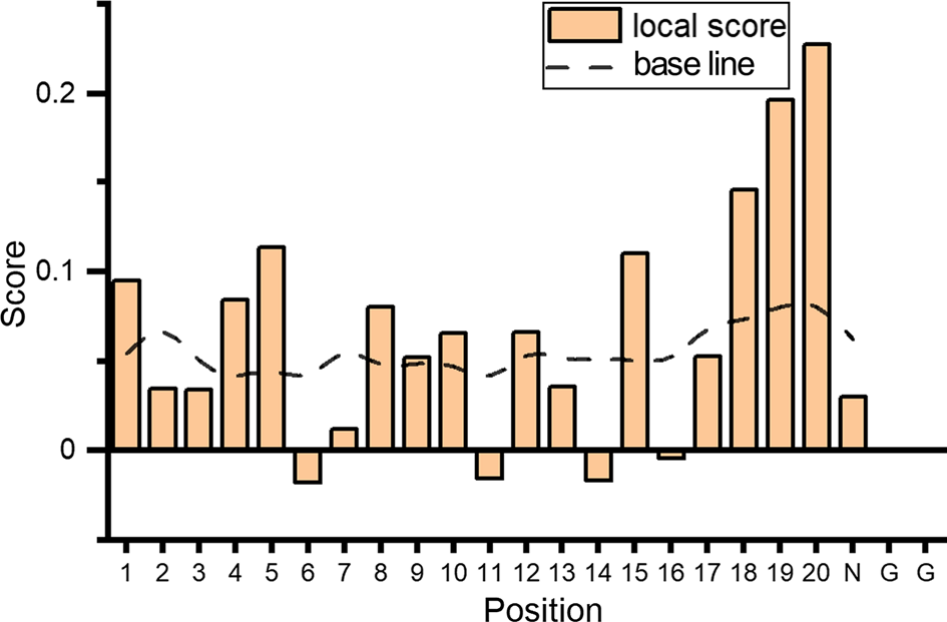
The scores of sgRNA with index of 8493 obtained by the temporal AttCRISPR. The bar plot reveals the score at each position, while the line of dashes reveals the average scores which can be used as a base line

For the first question, we input the least-active sgRNA in Additional file 1: Supplementary Tables Tab. S1 (with the index of 8493 and the activity of 0.831, call source sgRNA for convenience) into the temporal AttCRISPR. The score of each position is obtained based on Eq. (6) (the calculated symbol with *W* is Hadamard product instead of the dot product, to achieve the result in vector form), and the results are shown in Fig. 5 and Additional file 2: Data S4, in which scores at position 14 and 16 of source sgRNA are significantly below the base line (in fact, the scores at position 6 and 11 is also noteworthy, however, we don't find sgRNA in the dataset for comparison). If we replaced the T at position 14 with C, would generate the same sgRNA as the one with index of 8491, which is with an activity value of 0.861. If we replaced the T at position 16 with C, would generate the same sgRNA as the one with index of 8492, which is with an activity value of 0.869. Therefore, we could conclude that the local interpretability is helpful for us to optimize the sgRNA without exhaustive search.

**Fig. 6:**
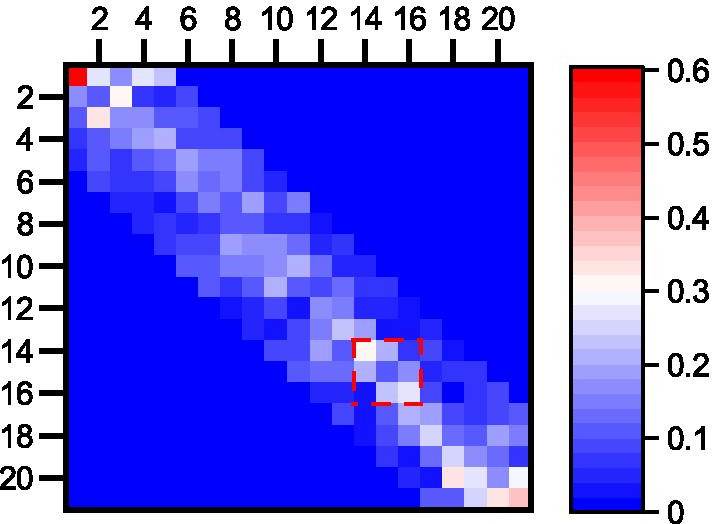
The visualization of the second-order preference matrix *B*, the elements in the *i*-th row and the *j*-th column represent the influence of nucleotide at position *j* when generating the first-order preference $\tilde{A}$ at position *i*. The warmer the color, the more important it is. In the red box, a few unusual bright spots appear. To be more specific, the nucleotide at position 15 has a great effect on the first-order preference at position 14 and 16

The second question we expect AttCRISPR to answer is why it gave two low scores at position 14 and 16. In practice, we will try to answer this question with second-order preference. Let AttCRISPR output the second-order preference matrix *B* corresponding to the source sgRNA, and show it in Fig. 6, a few unusual bright spots appear in the red box in Fig. 6, which show that the nucleotide at position 15 has a great effect on the score of position 14 and 16 (instead of position 13 or 17, which the corresponding position are relatively dim in color). As shown in Additional file 1: Supplementary Tables Tab. S1, in source sgRNA there are three consecutive Ts at position 14, 15 and 16, and this may reveal that multiple consecutive Us on sgRNA would lead to the low on-target activity of sgRNA, which is consistent with an earlier report [28].

## Discussion

In this article, we have developed a new prediction method, called AttCRISPR for the activity of sgRNA. We take the ensemble of both spatial and temporal domains to predict the on-target activity of sgRNA. Through ablation analysis and testing a series of possible network structures, we demonstrate that the ensemble method performs better than other methods on this task. In addition, we apply attention modules in both the spatial and temporal parts of AttCRISPR, and design two experiments combined with some early reports to prove that attention mechanisms can help researchers understand the decisions made by the model which makes it easy to optimize low activity sgRNA without exhaustive search.

As shown in Fig. 6, we note that the brightness at coordinates (14, 15) and (16, 15) exceeds (14, 13) and (16, 17). This could explain that the nucleotide trimer at positions 14, 15, 16 has a great influence on the decision made by AttCRISPR. We believe that we can use a carefully designed 3 × 3 convolution kernel, and move it along the diagonal of the second-order preference matrix *B*, in order to find all kinds of nucleotide trimer that have a great influence on the decision made by AttCRISPR. Further experiments may be needed for validation.

In addition, based on the attention modules and the given sgRNA activity data, researchers can optimize existing sgRNA through global and local nucleotide importance analysis results, to design highly active sgRNA.

The current architecture of AttCRISPR focuses on predicting the on-target activity of conventional sgRNA which have a PAM based on NGG. However, it can be extended to other Cas9 species, variants or off-target tasks easily.

## Conclusion

In this paper, we develop AttCRISPR, an ensemble of both spatial and temporal methods that follow the stacking strategy with strong interpretability. AttCRISPR proves that the ensemble methods have a better performance in the dataset of DeepHF and can compete with current state-of-the-art methods. In addition, AttCRISPR applies attention mechanisms in both the temporal and spatial parts, and we explain the decisions made by AttCRISPR through the attention module which is consistent with earlier reports. Further, we also discovered that the output of the attention module can be used to optimize the low-activity sgRNA without exhaustive search, and the optimization results are verified with available experimental data.

## Supplementary Information

**Additional file 1.** Overview of the supplemental information.

**Additional file 2.** Datasets used in the experiment.

## Data Availability

The example code is available at https://github.com/South-Walker/AttCRISPR.

## References

[1] Jinek M, Chylinski K, Fonfara I, Hauer M, Doudna JA, Charpentier E (2012). A programmable dual-RNA-guided DNA endonuclease in adaptive bacterial immunity. Science.

[2] Cong L, Ran FA, Cox D, Lin S, Barretto R, Habib N, Hsu PD, Wu X, Jiang W, Marraffini LA, Zhang F (2013). Multiplex genome engineering using CRISPR/Cas systems. Science.

[3] Mali P, Yang L, Esvelt KM, Aach J, Guell M, DiCarlo JE, Norville JE, Church GM (2013). RNA-guided human genome engineering via Cas9. Science.

[4] Rubeis G, Steger F (2018). Risks and benefits of human germline genome editing: an ethical analysis. Asian Bioeth Rev.

[5] Kang X, He W, Huang Y, Yu Q, Chen Y, Gao X, Sun X, Fan Y (2016). Introducing precise genetic modifications into human 3PN embryos by CRISPR/Cas-mediated genome editing. J Assist Reprod Genet.

[6] Ishii T (2017). Reproductive medicine involving genome editing: clinical uncertainties and embryological needs. Reprod Biomed Online.

[7] Liang P, Xu Y, Zhang X, Ding C, Huang R, Zhang Z, Lv J, Xie X, Chen Y, Li Y, Sun Y, Bai Y, Songyang Z, Ma W, Zhou C, Huang J (2015). CRISPR/Cas9-mediated gene editing in human tripronuclear zygotes. Protein Cell.

[8] Slaymaker IM, Gao L, Zetsche B, Scott DA, Yan WX, Zhang F (2016). Rationally engineered Cas9 nucleases with improved specificity. Science.

[9] Kleinstiver BP, Pattanayak V, Prew MS, Tsai SQ, Nguyen NT, Zheng Z, Joung JK (2016). High-fidelity CRISPR-Cas9 nucleases with no detectable genome-wide off-target effects. Nature.

[10] Chuai G, Ma H, Yan J, Chen M, Hong N, Xue D, Zhou C, Zhu C, Chen K, Duan B, Gu F, Qu S, Huang D, Wei J, Liu Q (2018). DeepCRISPR: optimized CRISPR guide RNA design by deep learning. Genome Biol.

[11] Liu G, Zhang Y, Zhang T (2019). Computational approaches for effective CRISPR guide RNA design and evaluation. Comput Struct Biotechnol J.

[12] Kim HK, Min S, Song M, Jung S, Choi JW, Kim Y, Lee S, Yoon S, Kim HH (2018). Deep learning improves prediction of CRISPR-Cpf1 guide RNA activity. Nat Biotechnol.

[13] Kim HK, Kim Y, Lee S, Min S, Bae JY, Choi JW, Park J, Jung D, Yoon S, Kim HH. SpCas9 activity prediction by DeepSpCas9, a deep learning-based model with high generalization performance. Sci Adv. 2019; 5(11):eaax9249. 10.1126/sciadv.aax9249 PMC6834390 31723604

[14] Song M, Kim HK, Lee S, Kim Y, Seo SY, Park J, Choi JW, Jang H, Shin JH, Min S, Quan Z, Kim JH, Kang HC, Yoon S, Kim HH (2020). Sequence-specific prediction of the efficiencies of adenine and cytosine base editors. Nat Biotechnol.

[15] Muhammad Rafid AH, Toufikuzzaman M, Rahman MS, Rahman MS (2020). CRISPRpred(seq): a sequence-based method for sgRNA on target activity prediction using traditional machine learning. BMC Bioinform.

[16] Liu Q, He D, Xie L (2019). Prediction of off-target specificity and cell-specific fitness of CRISPR-Cas system using attention boosted deep learning and network-based gene feature. PLoS Comput Biol.

[17] Wang D, Zhang C, Wang B, Li B, Wang Q, Liu D, Wang H, Zhou Y, Shi L, Lan F, Wang Y (2019). Optimized CRISPR guide RNA design for two high-fidelity Cas9 variants by deep learning. Nat Commun.

[18] Lin J, Wong KC (2018). Off-target predictions in CRISPR-Cas9 gene editing using deep learning. Bioinformatics.

[19] Zhang G, Dai Z, Dai X (2020). C-RNNCrispr: Prediction of CRISPR/Cas9 sgRNA activity using convolutional and recurrent neural networks. Comput Struct Biotechnol J.

[20] Liu Q, Cheng X, Liu G, Li B, Liu X (2020). Deep learning improves the ability of sgRNA off-target propensity prediction. BMC Bioinform.

[21] Ashish Vaswani, Noam Shazeer, Niki Parmar, Jakob Uszkoreit, Llion Jones, Aidan N. Gomez, Lukasz Kaiser, and Illia Polosukhin. Attention Is All You Need. arXiv preprint https://arxiv.org/abs/1706.03762, June 2017.

[22] Woo S, Park J, Lee JY (2018). CBAM: convolutional block attention module.

[23] Luong MT, Pham H, Manning CD (2015). Effective approaches to attention-based neural machine translation. Comput Sci.

[24] Wang S, Peng J, Ma J, Xu J (2016). Protein secondary structure prediction using deep convolutional neural fields. Sci Rep.

[25] Wang T, Wei JJ, Sabatini DM, Lander ES (2014). Genetic screens in human cells using the CRISPR/Cas9 system. Science.

[26] Wong N, Liu W, Wang X (2015). WU-CRISPR: characteristics of functional guide RNAs for the CRISPR/Cas9 system. Genome Biol.

[27] Doench JG, Hartenian E, Graham DB, Tothova Z, Hegde M, Smith I, Sullender M, Ebert BL, Xavier RJ, Root DE (2014). Rational design of highly active sgRNAs for CRISPR-Cas9-mediated gene inactivation. Nat Biotechnol.

[28] Wu X, Scott DA, Kriz AJ, Chiu AC, Hsu PD, Dadon DB, Cheng AW, Trevino AE, Konermann S, Chen S, Jaenisch R, Zhang F, Sharp PA (2014). Genome-wide binding of the CRISPR endonuclease Cas9 in mammalian cells. Nat Biotechnol.

