# Supplementary material for: AttCRISPR: a spacetime interpretable model for prediction of sgRNA on-target activity
Source: BMC Bioinformatics. 2021 Dec 13;22:589. doi: 10.1186/s12859-021-04509-6 (PMC8667445; doi:10.1186/s12859-021-04509-6)
Supplement: Supplementary file 1 — Additional file 1. Overview of the supplemental information. [file 12859_2021_4509_MOESM1_ESM.zip › Additional file1-Overview of the Supplemental Information.docx]

**Supplemental Information**

**Supplementary Data**

**Data S1. Source data.** It contains 55604, 58617, 56888 sgRNAs with activity (represented by insertion/deletion (indel)) for WT-SpCas9, eSpCas9(1.1) and SpCas9-HF1, respectively.

**Data S2. The performance of each model in ten independent experiments.** The model performance is expressed by Spearman correlation coefficient.

**Data S3. Output of spatial attention module at global level.** We input every sgRNA into the spatial AttCRISPR, to obtain the $A_{s}$ from the spatial attention module and take its mean value. Then we standardize it through Z-score.

**Data S4. The scores at each position of sgRNA with index of 8493 obtained by the temporal AttCRISPR.** We input this sgRNA into the temporal AttCRISPR, to obtain the $W\otimes\tilde{A}X_{e}^{T}$ from the spatial attention module, where $\otimes$ denotes element-wise multiplication.

**Data S5. Output of temporal attention module at local level.** We input the sgRNA with index of 8493 into the temporal AttCRISPR, to obtain the $B$ from the temporal attention module.

**Supplementary Figures**

**
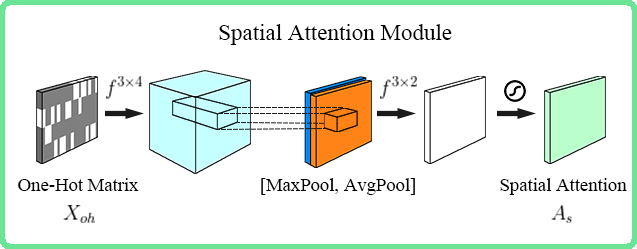
**

**Fig. S1. Details of spatial attention module.** A convolution layer is used to generate multi-channel map from $X_{oh}$. Then concatenated the output of both max-pooling and average-pooling method and forward it to the last convolution layer. A sigmoid function is used to map the final result to a range of zero to one at last, which generates the spatial first-order preference matrix $A_{s}$.


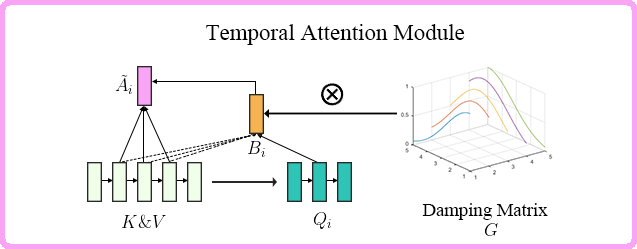


**Fig. S2. Details of temporal attention module.**

To generate the first order preference vector in the $i$ position of sgRNA $\tilde{A}_{i}$. First, the $i$ -th row vector of the queries matrix $Q_{i}$ is multiplied by the transpose of the keys matrix $K^{T}$, and apply a softmax function to obtain a preliminary weights vector on the values.

Second, to favor the alignment points near $i$, the weights vector obtained is multiplied element-by-element by the $i$ -th row vector of the damping matrix $G$. $G_{ij}$ can be regarded as the result of place a Gaussian distribution centered around $i$, then sampling the position $j$ (a scaling factor is used to ensure the sum of $G_{i}$ is 1).

Then we achieve the second-order preference matrix $B$, it is also a weights matrix on the values. So the first-order preference matrix $\tilde{A}$ come from the product of $B$ and values matrix $V$. Temporal attention module generates the temporal first-order preference matrix $\tilde{A}$ and the temporal second-order preference matrix $B$.





**Fig. S3. In the absence of hand-crafted biological features, performance of different algorithms for sgRNA activity prediction.**

(a)-(c) The performance of Temporal AttCRISPR, Spatial AttCRISPR and Ensemble AttCRISPR.

The half-violin plots show the mean and distribution of the Spearman correlation coefficient between predicted and measured sgRNA activity scores over all tests.

(d)-(f) In the absence of hand-crafted biological features, the performance of all prediction methods in these datasets as far as we know.

The $mean\pm s.d.$ of the Spearman correlation coefficient between predicted and measured sgRNA activity scores are shown in the bar plots.

**

**

**Fig. S4. Performance comparisons for the methods before and after integrating with hand-crafted biological features.**

where DeepHF is the RNN integrated with hand-crafted biological features, and StAC is the EnAC integrated with hand-crafted biological features. The box plot shows the mean and distribution of Spearman correlation coefficient between predicted and measured sgRNA activity scores over all tests.

**Supplementary Tables**

**Tab. S1. Three sgRNA and their activity.** Activity of sgRNA represents the activity reported in the WT-SpCas9 dataset (Supplementary Data 1).

| Index | sgRNA | Activity |
| --- | --- | --- |
| 8493 | ACATGACTTTGGATTTCCCCAGG | 0.831 |
| 8492 | ACATGACTTTGGATTCCCCCAGG | 0.869 |
| 8491 | ACATGACTTTGGACTTCCCCAGG | 0.861 |
